# Segmentation of glioblastomas via 3D FusionNet

**DOI:** 10.3389/fonc.2024.1488616

**Published:** 2024-11-15

**Authors:** Xiangyu Guo, Botao Zhang, Yue Peng, Feng Chen, Wenbin Li

**Affiliations:** Department of Neuro-Oncology, Cancer Center, Beijing Tiantan Hospital, Capital Medical University, Beijing, China

**Keywords:** brain tumor segmentation, MRI, U-net, SegNet, 3D deep learning model

## Abstract

**Introduction:**

This study presented an end-to-end 3D deep learning model for the automatic segmentation of brain tumors.

**Methods:**

The MRI data used in this study were obtained from a cohort of 630 GBM patients from the University of Pennsylvania Health System (UPENN-GBM). Data augmentation techniques such as flip and rotations were employed to further increase the sample size of the training set. The segmentation performance of models was evaluated by recall, precision, dice score, Lesion False Positive Rate (LFPR), Average Volume Difference (AVD) and Average Symmetric Surface Distance (ASSD).

**Results:**

When applying FLAIR, T1, ceT1, and T2 MRI modalities, FusionNet-A and FusionNet-C the best-performing model overall, with FusionNet-A particularly excelling in the enhancing tumor areas, while FusionNet-C demonstrates strong performance in the necrotic core and peritumoral edema regions. FusionNet-A excels in the enhancing tumor areas across all metrics (0.75 for recall, 0.83 for precision and 0.74 for dice scores) and also performs well in the peritumoral edema regions (0.77 for recall, 0.77 for precision and 0.75 for dice scores). Combinations including FLAIR and ceT1 tend to have better segmentation performance, especially for necrotic core regions. Using only FLAIR achieves a recall of 0.73 for peritumoral edema regions. Visualization results also indicate that our model generally achieves segmentation results similar to the ground truth.

**Discussion:**

FusionNet combines the benefits of U-Net and SegNet, outperforming the tumor segmentation performance of both. Although our model effectively segments brain tumors with competitive accuracy, we plan to extend the framework to achieve even better segmentation performance.

## Introduction

Brain is an organ that functions as the central hub of the nervous system, composed of various cell types and a complex microenvironment. According to the Global Burden of Disease Study 2019, brain and central nervous system tumors are among the most common types of cancers, with an estimated 350,639 cases and 252,814 cancer-related deaths in 2019 (1). Due to intricated biology and microenvironment of the brain, each type of tumor has its own characteristics, posing a challenge for clinicians to accurately describe the classification, location, and size of tumors (2, 3). Medical experts usually detect brain tumors through neurological exams, imaging practices, and biopsies. Among imaging tests, Magnetic Resonance Imaging (MRI) plays a crucial role in identifying the lesion location, extent of tissue involvement, and the resultant mass effect on the brain, ventricular system, and vasculature, due to its superior performance in visualizing organs and soft tissues (4, 5). Compared to regular X-rays and CT scans, MRI provides clearer images of non-bony parts such as the brain, spinal cord, nerves, and muscles. Since MRI does not use X-rays or other radiation, it is the preferred imaging test when frequent imaging is required for diagnosis or therapy, especially for brain imaging.

Gliomas, one of the most common types of brain cancer, often intermingle with healthy brain tissue and develop within the substance of the brain. Glioblastoma (GBM) constitutes the majority of WHO grade 4 gliomas and is one of the most lethal and recurrence-prone malignant solid tumors, accounting for 57% of all gliomas and 48% of primary central nervous system malignant tumors (6). Because of its unclear morphological structures, it is challenging for physicians to accurately identify the lesion location, extent of tissue involvement, and level of malignancy. Therefore, a series of computer-aided diagnostic (CAD) tools have been applied to assist in the more accurate diagnosis of cancer (7–9).

Tumor segmentation, the process of accurately separating tumors from their background, is one of the crucial steps in radiomics, diagnosis and treatments of brain tumors (10, 11). With the growing demands of clinical applications and scientific research, image segmentation has become increasingly important in the field of medical imaging processing. However, fully manual medical image segmentation is time-consuming and requires expertise. The advancement of CAD techniques has made it easier to complete tasks more efficiently. Image segmentation can be performed using numerous techniques, ranging from conventional methods to advanced deep learning approaches. In traditional techniques, the most common types are thresholding, region-based segmentation, edge-based segmentation, and clustering (12–18). Various machine learning methods, such as support vector machines, have also been effectively applied to medical imaging segmentation (19, 20). The emergence of deep learning has markedly boosted the precision and speed of image segmentation processes. These deep learning methods include generative adversarial networks (GANs), recurrent neural networks (RNNs), diffusion models and convolutional neural networks (CNNs) (21–25). Of the different deep learning techniques, CNNs continue to be the most widely used method for brain tumor segmentation (26). A study utilized a cascaded 3D Fully Convolutional Network (FCN) to automatically detect and segment brain metastases with high accuracy. This method was also effective in distinguishing brain metastases from high-grade gliomas (27). U-Net and SegNet are two commonly used network architectures for semantic segmentation. Skip connections in U-Net aid in better recovering details and boundary information in segmentation tasks (28). Conversely, SegNet uses pooling indices for upsampling, which may lose some details but offers lower memory consumption and higher computational efficiency (29). U-Net 3+ incorporates multiple improvements over the U-Net structure, enabling it to better capture image details and contextual information, making it suitable for more complex and diverse segmentation tasks (30, 31).

In this study, to fully utilize the advantages of both networks, we developed a 3D FusionNet that combines the features of U-Net and SegNet. This end-to-end trainable model aims to achieve better segmentation performance in medical images.

## Methods

### Data collection

The MRI data used in this study were obtained from a cohort of 630 GBM patients from the University of Pennsylvania Health System (UPENN-GBM) (32). This dataset, which includes imaging data, clinical data, and radiomic data, is available through the Cancer Imaging Archive (TCIA) at the National Cancer Institute (33, 34). All MRI data were obtained prior to surgery using 3T MRI scanners and included T1, T2, ceT1, FLAIR, and related segmentation labels. Of the 630 patients, 611 with complete MRI data were used in this study. The MRI data consist of Glioblastoma patient scans, meticulously annotated by clinical experts to highlight sub-regions such as the necrotic core (NC), peritumoral edema (ED), and enhancing tumor (ET).

### Data augmentation

The MRI data from UPENN-GBM were already well-preprocessed, so no additional preprocessing steps were performed. To better adapt to the network structure, we applied zero-padding to the images before data augmentation, expanding their sizes from the original 240*240*155 voxels to 256*256*256 voxels. We selected 80 samples from the 611 as the test set, 31 samples as the validation set, and the remaining samples as the training set. Subsequently, we employed a series of data augmentation techniques, including horizontal flip, vertical flip, and 90-, 180-, and 270-degrees rotations, to further increase the sample size of the training set to 3,000 samples.

### Proposed network

**Figure 1:**
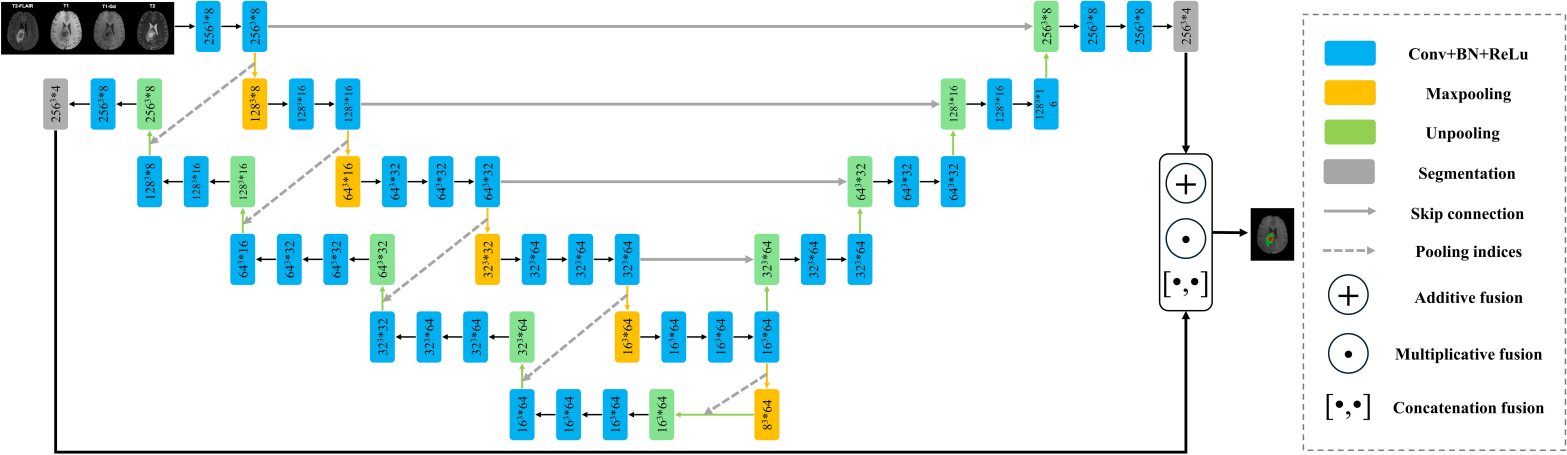
The network structure of FusionNet. Note: Here, for example, 2563*8 indicates that the feature map size is 256, and the channel count is 8. The symbol * represents multiplication.

The proposed end-to-end network architecture is shown in **Figure 1**. It comprises the following components: a shared encoder with 13 convolutional layers, mirroring the convolutional layers of the VGG16 network; two distinct decoders, one for U-Net and another for SegNet; and an optional joint output layer for the two decoders, offering additive (A), elementwise multiplicative (M), and concatenation (C) operations. Unlike traditional ensemble learning methods, our approach employs a shared encoder that enhances parameter efficiency, feature reusability, and model generalization. This design simplifies the architecture and promotes stable training by enabling both decoders to leverage the same robust feature extraction process. The additive (A) operation sums the outputs from the two decoders, harnessing the strengths of both architectures to enhance overall feature representation. The elementwise multiplicative (M) operation multiplies the outputs from the decoders elementwise, allowing for a more nuanced interaction between their outputs and effectively emphasizing areas of consensus. Finally, the concatenation (C) operation merges the outputs along the channel dimension, preserving the unique features from both decoders and enabling the model to leverage diverse representations.

Given the 3D structure of both the volumetric data and the network, which demands substantial computational memory, we had to reduce the number of channels in the convolutional layers. The specific sizes and channels of the feature maps at various stages are illustrated in **Figure 1**.

The loss function employed in this network is cross-entropy loss, which measures voxel-wise similarity between the prediction and ground truth. Each voxel in the image is treated as an independent classification task, and the cross-entropy loss is computed across all voxels in the image, as show in the Equation 1:


(1) $$\begin{array}{c} L=-\frac{1}{H*W*D}\sum_{h=1}^{H}\sum_{w=1}^{W}\sum_{d=1}^{D}\sum_{c=1}^{C}y_{h,w,d,c}\log\left({\hat{y}}_{h,w,d,c}\right) \end{array}$$

where $y_{h,w,d,c}$ is the true label for the pixel at position (h,w,d) for class c , ${\hat{y}}_{h,w,d,c}$ is the predicted probability for voxel (h,w,d) belonging to class c , given by the model. The loss is averaged over all voxels to obtain a single scalar value representing the loss for the entire image.

### Perfomance metrics

In this study, the segmentation performance of models was evaluated by recall, precision, dice score, Lesion False Positive Rate (LFPR), Average Volume Difference (AVD) and Average Symmetric Surface Distance (ASSD) (35–37). Recall measures the ability of the model to correctly identify positive instances as show in the Equation 2.


(2) $$\begin{array}{c} Recall=\frac{TP}{TP+FN} \end{array}$$

Precision measures the accuracy of the positive predictions as show in the Equation 3.


(3) $$\begin{array}{c} Precision=\frac{TP}{TP+FP} \end{array}$$

Dice Score is the harmonic mean of precision and recall, providing a single metric to evaluate the balance between the two as show in the Equation 4.


(4) $$\begin{array}{c} Dice=\frac{2*TP}{TP+FN+TP+FP} \end{array}$$

Lesion False Positive Rate (LFPR) measures the proportion of non-lesion areas mistakenly identified as lesions by the model as show in the Equation 5. The LFPR values are small because the background occupies a large proportion. Due to the excessively low LFPR values, we performed normalization.


(5) $$\begin{array}{c} LFPR=\frac{FP}{FP+TN} \end{array}$$

Average Volume Difference (AVD) assesses the difference in volume between the predicted lesions and the ground truth lesions as show in the Equation 6.


(6) $$\begin{array}{c} AVD=\frac{1}{N}\sum_{i=1}^{N}\left|V_{pred,i}-V_{true,i}\right|/V_{true,i} \end{array}$$

Average Symmetric Surface Distance (ASSD) quantifies the average distance between the surfaces of the predicted and ground truth lesions as show in the Equation 7.


(7) $$\begin{array}{c} ASSD=\frac{1}{2}\left(\frac{1}{N_{pred}}\sum_{p\in P}d\left(p,G\right)+\frac{1}{N_{true}}\sum_{g\in G}d\left(g,P\right)\right) \end{array}$$

True Positive (TP) means the number of instances correctly predicted as positive; false positive (FP) means the number of instances incorrectly predicted as positive; true negative (TN) means the number of instances correctly predicted as negative; false negative (FN) means the number of instances incorrectly predicted as negative; N represents the number of lesion regions; $V_{pred,i}$ denotes the volume of the i-th predicted region; $V_{pred,i}$ indicates the volume of the i-th ground truth region; $N_{pred}$ represents the number of points on the predicted surface; $N_{true}$ represents the number of points on the ground truth surface; d(p,G) refers to the distance from each point on the predicted surface to the nearest point on the ground truth surface; while d(g,P) signifies the distance from each point on the ground truth surface to the nearest point on the predicted surface.

## Results

### Implementation details

In this study, we utilized four standard MRI modalities along with their corresponding expert-annotated segmentation maps to train the network. Both training and testing were conducted on an 80 GB NVIDIA A100 Tensor Core GPU using the Torch backend. The model’s initial parameters were obtained through PyTorch’s default initialization, and the network parameters were updated using the stochastic gradient descent optimization algorithm. We divided the 611 samples from UPENN-GBM into a training set (500 samples, 82%), which was augmented to reach a total of 3,000 samples, a validation set (31 samples, 5%), and a test set (80 samples, 13%). Specifically, the learning rate decays exponentially, with an initial value set to 0.01 and a decay factor of 0.8. The training was conducted over 20 epochs with a batch size of 5.

### Segmentation performance with different methods

First, we compared the segmentation performance of the proposed network with U-Net, SegNet, and U-Net3+ using FLAIR, T1, ceT1, and T2 MRI modalities. Due to limited computational memory, U-Net3+ with reduced channels performed poorly and was not included in the results table. Overall, the models were more likely to accurately segment peritumoral edema regions and enhancing tumor areas compared to necrotic core regions. In terms of FusionNet, recall, precision, and dice scores for peritumoral edema regions and enhancing tumor areas generally achieve around 0.75 to 0.80. However, the metrics for necrotic core regions were only around 0.50. As shown in **Table 1A**, our proposed method, which combines the benefits of U-Net and SegNet, achieved comparable tumor segmentation performance to both U-Net and SegNet in terms of recall, precision, and dice scores. U-Net performs best in recall (0.66) for the necrotic core regions but fails in the enhancing tumor areas. SegNet does not perform best in any region or metric. FusionNet-A excels in the enhancing tumor areas across all metrics (0.75 for recall, 0.83 for precision and 0.74 for dice scores) and also performs well in the peritumoral edema regions (0.77 for recall, 0.77 for precision and 0.75 for dice scores). FusionNet-M has good performance in the peritumoral edema regions (0.77 for recall, 0.70 for precision and 0.70 for dice scores) but does not lead in any specific metric. FusionNet-C has balanced performance, achieving the best precision and dice scores in the necrotic core regions (0.60 for precision and 0.54 for dice scores) and the best precision in the peritumoral edema regions (0.81). In summary, when applying FLAIR, T1, ceT1, and T2 MRI modalities, FusionNet-A and FusionNet-C emerged as the top-performing models overall. FusionNet-A particularly excelled in the segmentation of enhancing tumor areas, while FusionNet-C demonstrated strong performance in segmenting the necrotic core and peritumoral edema regions. U-Net has the best recall for the necrotic core region but lacks in other areas. SegNet only performs reasonably well in the segmentation of peritumoral edema regions but is significantly worse than the other models overall.

**Table 1A:** Summary of commonality performance metrics for different methods.

Methods	Recall			Precision			Dice		
	NC	ED	ET	NC	ED	ET	NC	ED	ET
U-Net	0.66 ± 0.30	0.75 ± 0.17	0	0.28 ± 0.18	0.71 ± 0.21	0.12 ± 0.24	0.33 ± 0.18	0.72 ± 0.19	0
SegNet	0	0.36 ± 0.16	0	0	0.51 ± 0.24	0	0	0.40 ± 0.18	0
FusionNet-A	0.43 ± 0.25	**0.77 ± 0.18**	**0.75 ± 0.26**	0.57 ± 0.24	0.77 ± 0.17	**0.83 ± 0.19**	0.45 ± 0.23	**0.75 ± 0.17**	**0.74 ± 0.24**
FusionNet-M	0.05 ± 0.04	0.77 ± 0.22	0.71 ± 0.29	0.40 ± 0.27	0.70 ± 0.23	0.78 ± 0.13	0.08 ± 0.06	0.70 ± 0.23	0.70 ± 0.24
FusionNet-C	0.54 ± 0.22	0.71 ± 0.21	0.70 ± 0.25	**0.60 ± 0.24**	**0.81 ± 0.17**	0.75 ± 0.16	**0.54 ± 0.21**	0.74 ± 0.19	0.69 ± 0.20

The evaluated metrics are presented as mean ± standard deviation. NC, Necrotic Core; ED, Peritumoral Edema; ET, Enhancing Tumor. Bold values indicate the best value for each specific metric within each category.

**Table 1B:** Summary of differences in performance metrics for different methods.

Methods	LFPR			AVD	ASSD		
	NC	ED	ET		NC	ED	ET
U-Net	0.04 ± 0.03	0.03 ± 0.03	0	1.35 ± 0.94	1.81 ± 1.43	1.24 ± 1.81	13.07 ± 4.53
SegNet	0	0.04 ± 0.02	0	0.80 ± 0.08	Nan	2.36 ± 2.18	Nan
FusionNet-A	0.01 ± 0.02	0.02 ± 0.02	0.02 ± 0.12	0.46 ± 1.01	2.02 ± 2.91	1.22 ± 2.19	1.68 ± 5.82
FusionNet-M	0	0.05 ± 0.12	0.01 ± 0.01	0.55 ± 0.38	2.95 ± 3.26	2.56 ± 6.09	1.15 ± 2.52
FusionNet-C	**0.01 ± 0.01**	**0.02 ± 0.02**	**0.01 ± 0.01**	**0.34 ± 0.31**	**1.31 ± 1.55**	**0.94 ± 1.72**	**0.85 ± 1.21**

The evaluated metrics are presented as mean ± standard deviation. LFPR, Lesion False Positive Rate; AVD, Average Volume Difference; ASSD, Average Symmetric Surface Distance; NC, Necrotic Core; ED, Peritumoral Edema; ET, Enhancing Tumor. Bold values indicate the best value for each specific metric within each category.

On the other hand, we compared different models based on LFPR, AVD, and ASSD (**Table 1B**). In terms of LFPR, FusionNet models significantly outperformed U-Net and SegNet across all three sub-regions. Among the FusionNet models, FusionNet-C excelled in segmenting the necrotic core, peritumoral edema regions, and enhancing tumor areas, achieving values of 0.01, 0.02, and 0.01, respectively. Additionally, FusionNet-C demonstrated the best AVD index, with a value of 0.34. Moreover, it achieved the best ASSD scores across all three sub-regions, with 1.31 for the necrotic core, 0.94 for peritumoral edema, and 0.85 for enhancing tumor regions, highlighting its superior overall segmentation performance.

### Segmentation performance with different combinations of MRI modalities

Next, we assessed the effectiveness of segmentation results from various combinations of MRI modalities based on FusionNet-A. As shown in **Table 2**, reasonable segmentation results can be achieved using various combinations of MRI modalities. Notably, we found that FLAIR and ceT1 are crucial components in segmentation tasks. Combinations including FLAIR and ceT1 tend to have better segmentation performance, especially for necrotic core regions. Additionally, using either FLAIR or ceT1 alone can still yield somewhat satisfactory results, although slightly less effective compared to combinations of multiple modalities. From **Table 2**, we can see that using only FLAIR achieves a recall of 0.73 for peritumoral edema regions, and using only ceT1 achieves a recall of 0.50 for necrotic core regions (the best recall for necrotic core regions among all combinations of MRI modalities is 0.51). On the other hand, some combinations of two or three MRI modalities also achieve better performance in specific metrics than using all four MRI modalities. Using FLAIR, T1 and ceT1 results in the best recall of 0.81 for enhancing tumor regions, the best precision of 0.81 for peritumoral edema regions and the best dice scores of 0.77 for peritumoral edema regions among all combinations of MRI modalities. The combination of FLAIR, ceT1, and T2 achieves the best recall of 0.51 for necrotic core regions and the best precision of 0.89 for enhancing tumor regions. The combination of T1, ceT1, and T2 achieves the best precision of 0.63 for necrotic core regions. Using FLAIR and ceT1 achieves the best recall of 0.84 for peritumoral edema regions among all combinations of MRI modalities. Another interesting finding was that combinations with T1 might reduce segmentation performance in necrotic core and enhancing tumor regions. Using T1 alone or in combinations without ceT1, metrics for necrotic core and enhancing tumor regions were significantly lower than others, sometimes even falling below 0.10.

**Table 2:** Summary of performance metrics for different combinations of MRI modalities based on FusionNet-A.

Modalities	Recall			Precision			Dice		
	NC	ED	ET	NC	ED	ET	NC	ED	ET
FLAIR/T1/T1c/T2	0.43 ± 0.25	0.77 ± 0.18	0.75 ± 0.26	0.57 ± 0.24	0.77 ± 0.17	0.83 ± 0.19	**0.45 ± 0.23**	**0.75 ± 0.17**	0.74 ± 0.24
FLAIR/T1/T1c	0.42 ± 0.25	0.71 ± 0.20	**0.81 ± 0.23**	0.58 ± 0.26	**0.81 ± 0.18**	0.80 ± 0.18	0.44 ± 0.24	0.73 ± 0.18	**0.77 ± 0.23**
FLAIR/T1/T2	0.12 ± 0.17	**0.83 ± 0.15**	0.07 ± 0.10	0.56 ± 0.33	0.53 ± 0.19	0.39 ± 0.24	0.17 ± 0.20	0.63 ± 0.19	0.10 ± 0.11
FLAIR/T1c/T2	**0.51 ± 0.27**	0.70 ± 0.17	0.49 ± 0.29	0.48 ± 0.25	0.72 ± 0.21	**0.89 ± 0.15**	0.44 ± 0.25	0.70 ± 0.18	0.57 ± 0.29
T1/T1c/T2	0.35 ± 0.25	0.68 ± 0.22	0.74 ± 0.25	**0.63 ± 0.26**	0.69 ± 0.19	0.80 ± 0.21	0.40 ± 0.25	0.66 ± 0.19	0.73 ± 0.24
FLAIR/T1	0.04 ± 0.06	0.74 ± 0.18	0.14 ± 0.16	0.38 ± 0.27	0.56 ± 0.22	0.44 ± 0.25	0.07 ± 0.09	0.62 ± 0.21	0.17 ± 0.18
FLAIR/T1c	0.34 ± 0.25	**0.84 ± 0.18**	0.58 ± 0.30	0.53 ± 0.25	0.64 ± 0.18	**0.84 ± 0.21**	0.35 ± 0.23	**0.70 ± 0.17**	0.61 ± 0.27
FLAIR/T2	0.39 ± 0.23	0.65 ± 0.17	0.33 ± 0.19	0.38 ± 0.22	0.64 ± 0.22	0.45 ± 0.19	0.34 ± 0.21	0.63 ± 0.18	0.32 ± 0.16
T1/T1c	0.35 ± 0.26	0.33 ± 0.21	0.69 ± 0.29	0.50 ± 0.26	0.54 ± 0.27	0.79 ± 0.26	0.36 ± 0.23	0.37 ± 0.21	**0.70 ± 0.27**
T1/T2	0.09 ± 0.16	0.66 ± 0.18	0.18 ± 0.17	0.30 ± 0.32	0.50 ± 0.21	0.32 ± 0.22	0.11 ± 0.18	0.55 ± 0.20	0.19 ± 0.15
T1c/T2	**0.45 ± 0.26**	0.55 ± 0.19	**0.76 ± 0.21**	**0.61 ± 0.27**	**0.75 ± 0.21**	0.73 ± 0.21	**0.47 ± 0.24**	0.61 ± 0.20	**0.70 ± 0.18**
FLAIR	0.23 ± 0.16	**0.73 ± 0.17**	0.28 ± 0.18	0.31 ± 0.17	**0.63 ± 0.22**	0.50 ± 0.21	0.22 ± 0.14	**0.66 ± 0.19**	0.31 ± 0.18
T1	0	0.15 ± 0.14	0	0.24 ± 0.37	0.34 ± 0.31	0.14 ± 0.25	0.01 ± 0.01	0.19 ± 0.18	0.01 ± 0.01
T1c	**0.50 ± 0.30**	0.33 ± 0.18	**0.57 ± 0.30**	**0.46 ± 0.26**	0.58 ± 0.27	**0.83 ± 0.25**	**0.40 ± 0.23**	0.38 ± 0.21	**0.62 ± 0.27**
T2	0.30 ± 0.28	0.54 ± 0.23	0.08 ± 0.09	0.37 ± 0.31	0.52 ± 0.24	0.32 ± 0.23	0.26 ± 0.24	0.51 ± 0.23	0.11 ± 0.10

The evaluated metrics are presented as mean ± standard deviation. NC, Necrotic Core; ED, Peritumoral Edema; ET, Enhancing Tumor. Bold values indicate the best value for each specific metric within each category.

### Visualization of brain tumor segmentation

**Figure 2:**
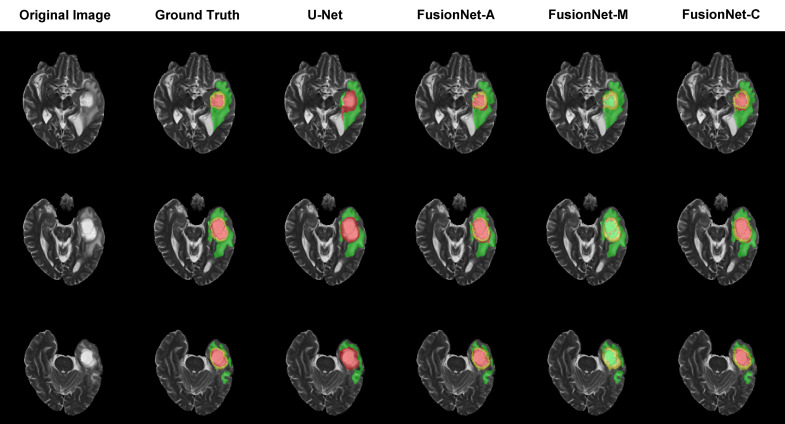
Segmentation results from a randomly selected subject in the axial plane. The first column shows the ground truths, the second column displays the original images, and the third column presents the FusionNet-A predicted segmentation results. Each row corresponds to different slices of T2 MRI images. Red indicates necrotic core regions, yellow represents enhancing tumor regions, and green denotes peritumoral edema regions.

**Figure 3:**
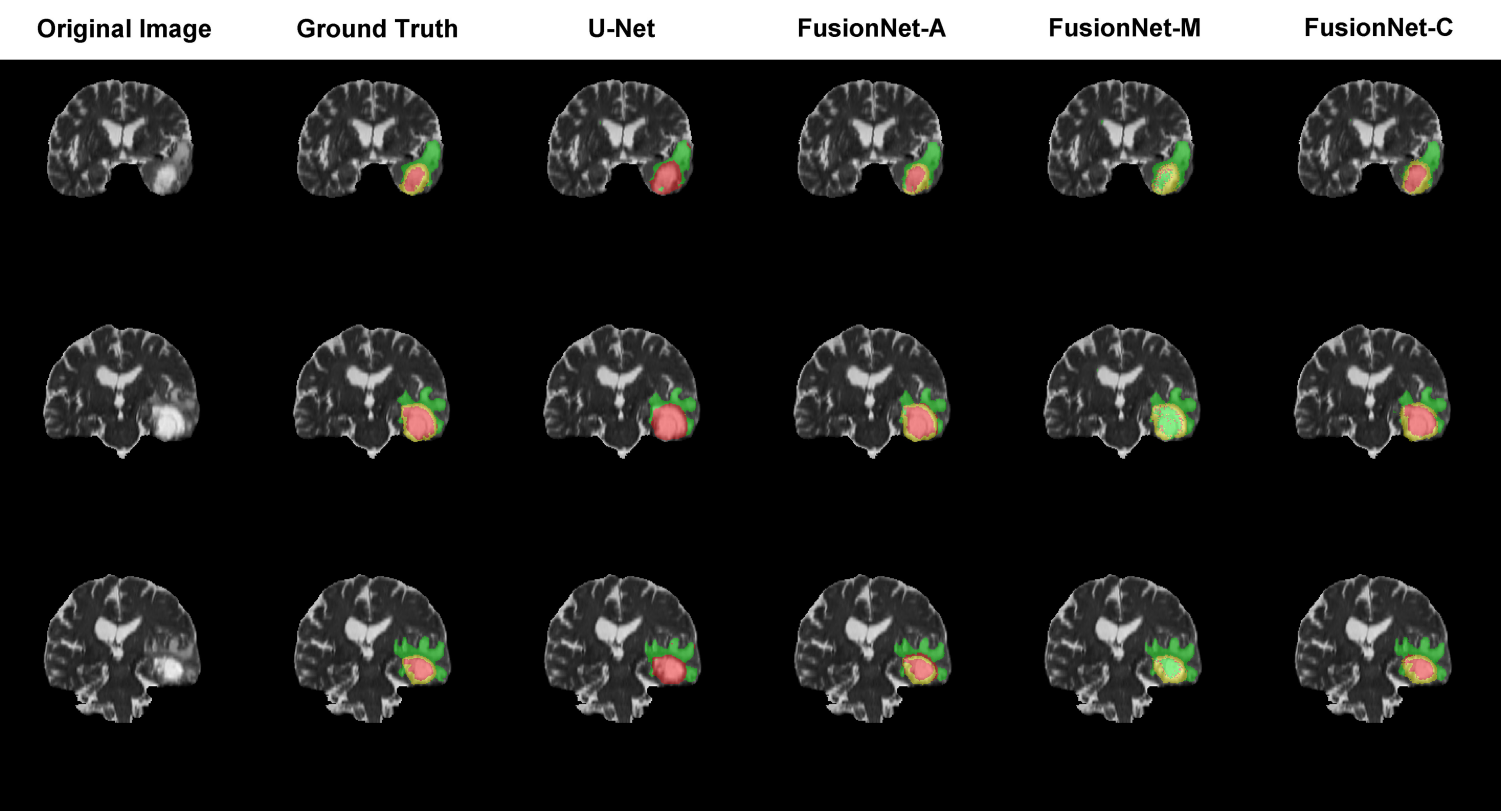
Segmentation results from a randomly selected subject in the coronal plane. The first column shows the ground truths, the second column displays the original images, and the third column presents the FusionNet-A predicted segmentation results. Each row corresponds to different slices of T2 MRI images. Red indicates necrotic core regions, yellow represents enhancing tumor regions, and green denotes peritumoral edema regions.

**Figure 4:**
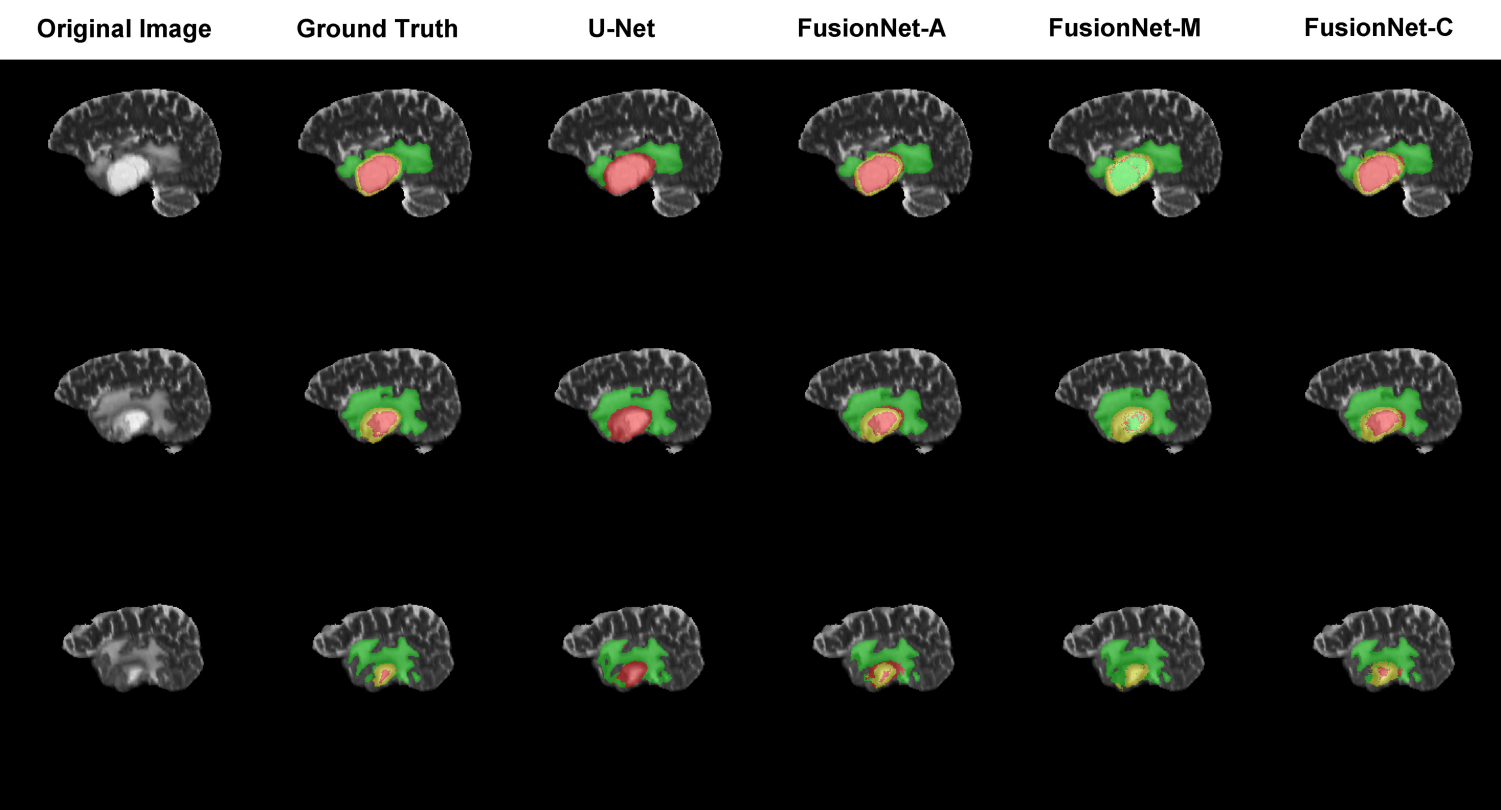
Segmentation results from a randomly selected subject in the sagittal plane. The first column shows the ground truths, the second column displays the original images, and the third column presents the FusionNet-A predicted segmentation results. Each row corresponds to different slices of T2 MRI images. Red indicates necrotic core regions, yellow represents enhancing tumor regions, and green denotes peritumoral edema regions.

Moreover, we visualized the brain tumor segmentation results from various models, excluding SegNet due to its comparatively poor performance relative to the other models. We randomly selected a subject’s T2 MRI modality with various colors representing different tumor regions. **Figures 2**–**4** show T2 MRI modality slices from the axial, coronal, and sagittal planes, respectively. In these figures, red indicates necrotic core regions, yellow represents enhancing tumor regions, and green denotes peritumoral edema regions. From left to right, the images display the original T2 image, the ground truth, and the segmentation results from U-Net, FusionNet-A, FusionNet-M and FusionNet-C. U-Net shows poor segmentation performance in the enhancing tumor areas, mistakenly classifying some of these regions as part of the necrotic core. This misclassification contributes to U-Net achieving the best recall for the necrotic core regions. Conversely, FusionNet-M tends to mistakenly identify necrotic core regions as peritumoral edema or enhancing tumor areas, leading to less precise segmentation in those regions. FusionNet-A and FusionNet-C generally achieve segmentation results that closely resemble the ground truth, although there are slight discrepancies in the segmentation of necrotic core regions compared to the manual annotations.

## Discussion

Brain tumors are among the most lethal diseases in humans. MRI images are a crucial diagnostic tool for identifying tumors by providing detailed pictures of soft tissues. Advanced methods for identifying and quantifying tumor lesions are vital in cancer treatment. Image analysis, one of these methods, offers critical information, such as lesion location, volume, count, extent of tissue involvement, and the resultant mass effect on the brain. A variety of state-of-art machine learning and deep learning models have been developed to automate the process of segmentation and classification of tumors. Many deep learning methods primarily focus on handling two-dimensional slices, which require less GPU memory and computational resources, and benefit from abundant available pre-trained weights (38–41). Compared to 2D models, 3D models use entire 3D MRI volumes as inputs, fully utilize 3D spatial information, better maintain the integrity of 3D structures, and provide more coherent segmentation results, but they require more GPU memory, computational resources, and longer training times.

In this study, we proposed a 3D end-to-end FusionNet for brain tumor segmentation using multiple MRI modalities, capable of adaptively handling an arbitrary number of modalities. We extensively explored the segmentation performance of our model compared to other segmentation methods using MRI data from UPENN-GBM. We utilize recall, precision, Dice score, LFPR, AVD, and ASSD to evaluate the segmentation performance, capturing both commonalities and differences between the predicted segmentation and the ground truth from two dimensions: overlap-based and distance-based metrics. FusionNet combines the benefits of U-Net and SegNet, achieving comparable tumor segmentation performance to both in terms of recall, precision, and dice scores with three different joint strategies. Overall, FusionNet-A and FusionNet-C are the best-performing models in overlap-based metrics, with FusionNet-A particularly excelling in the enhancing tumor areas, while FusionNet-C demonstrates strong performance in the necrotic core and peritumoral edema regions. Additionally, FusionNet-A and FusionNet-C also outperform in distance-based metrics, with FusionNet-C showing a slight edge over FusionNet-A. We observed that some models with poor performance in recall or Dice score had LFPR values approaching zero. This indicates that these models were unable to effectively segment the lesion areas, resulting in almost no false positive outcomes. Although FusionNet achieves comparable tumor segmentation results, its performance is limited due to the constraints on channels and layers. The input image size of 256*256*256 voxels generates numerous large feature maps during the learning process. Consequently, with limited memory resources available for abundant weights, we had to reduce the number of channels and layers. In future work, we will focus on balancing the feature map size with the number of channels and layers.

Compared to the performance of models on the Brain Tumor Segmentation (BraTS) dataset, our results using the UPENN-GBM dataset show lower index values (42, 43). While UPENN-GBM primarily focuses on glioblastoma (GBM), the BraTS dataset includes a wider variety of brain tumors, such as low-grade and high-grade gliomas. Glioblastoma presents additional challenges due to its indistinct boundaries and the unclear separation between its sub-regions (44). This blending with surrounding brain tissue makes segmentation tasks particularly difficult, which contributes to the lower performance indices observed in our experiments.

Additionally, we investigated segmentation performance with different combinations of MRI modalities. FLAIR and ceT1 are vital modalities for segmentation tasks; using either FLAIR or ceT1 alone or in combination tends to result in better segmentation performance, especially for necrotic core regions. However, combinations involving T1 may reduce segmentation performance in necrotic core and enhancing tumor regions. FLAIR, T1, ceT1, and T2 MRI modalities each have unique characteristics for glioma segmentation tasks. The FLAIR sequence is particularly sensitive to peritumoral edema region, presenting as high signal intensity, and assists in segmenting the necrotic core area. The ceT1 sequence, after contrast agent injection, clearly shows the enhancing tumor region, significantly improving the segmentation of the necrotic core. The T1 sequence, without enhancement, has lower contrast for the necrotic core and enhancing tumor, resulting in poorer segmentation performance. The T2 sequence is sensitive to tissues with high water content, prominently highlighting the edema region and aiding in the segmentation of both the necrotic core and enhancing tumor. We will explore assigning different weights to the four sequences and organically combining them to achieve more accurate segmentation and evaluation of the different subregions in the future work.

To provide a more intuitive understanding of our model’s segmentation effect, we visualized the segmentation results of models alongside the corresponding original image and ground truth segmentation from the axial, coronal, and sagittal planes, respectively. FusionNet-A and FusionNet-C generally achieve segmentation results that closely resemble the ground truth compared to other models, although it performs slightly worse in the necrotic core regions. There are several reasons for this. First, the borders between the necrotic core, enhancing tumor, and peritumoral edema tissues are usually diffused and not clearly defined. Second, the model learns from training samples of original MRI images and their corresponding ground truth annotations, which were made by different people. Due to the unclear borders, each annotator may have a slightly different interpretation of the boundaries between the different tumor subregions. Lastly, global segmentation methods, such as semantic segmentation models, are prone to being influenced by background noise, especially when it comes to segmenting small targets, where they often underperform. This is because, in the process of handling the entire image, the features of small targets can easily be overshadowed or diminished by background information, making it difficult for the model to accurately capture the boundaries and shapes of these regions.

Our study has some limitations. First, as a 3D model, FusionNet requires more GPU memory and computational resources. Due to the higher computational cost and large feature maps, FusionNet must have limited channels and layers compared to its 2D counterpart, which might constrain the performance of the model. Second, the current model has slightly worse segmentation performance on necrotic core regions compared to the other two regions. To address the challenge of limited computing resources and poor performance for small targets, we will explore two approaches in future works. First, we will investigate the impact of different MRI image sizes on segmentation model performance, aiming to strike a reasonable balance between model depth and computational efficiency. Second, we will employ region-based segmentation, which involves initially locating and outlining the entire brain tumor, followed by segmenting the tumor sub-regions within this smaller, specific area.

The necrotic core (NC), enhancing tumor (ET), and peritumoral edema (ED) regions are of significant clinical importance (45, 46). The necrotic core is often associated with high-grade malignancies and poor prognosis. The enhancing tumor region indicates a disrupted blood-brain barrier and neovascularization, making it a key target for surgery and radiotherapy. The peritumoral edema reflects the tumor’s pressure on surrounding brain tissue, leading to clinical symptoms and impacting treatment planning. Assessing these regions is crucial for diagnosis, treatment decisions, and prognosis in brain tumor management. Although manual segmentation can yield highly accurate results, it is time-consuming and labor-intensive, especially in large-scale studies. Automated segmentation methods can effectively address these limitations, allowing for manual correction to ensure accuracy while significantly improving segmentation efficiency.

In this paper, we presented an end-to-end deep learning model for the automatic segmentation of brain tumors. Our model effectively segments brain tumors with competitive accuracy. However, brain tumor segmentation remains a challenging task, and we will further extend the framework of this model to achieve better segmentation performance.

## Data Availability

The original contributions presented in the study are included in the article/supplementary material. Further inquiries can be directed to the corresponding author.
